# Automatic classification of signal regions in ^1^H Nuclear Magnetic Resonance spectra

**DOI:** 10.3389/frai.2022.1116416

**Published:** 2023-01-11

**Authors:** Giulia Fischetti, Nicolas Schmid, Simon Bruderer, Guido Caldarelli, Alessandro Scarso, Andreas Henrici, Dirk Wilhelm

**Affiliations:** ^1^Dipartimento di Scienze Molecolari e Nanosistemi, Ca' Foscari Università di Venezia, Venice, Italy; ^2^Zürcher Hochschule für Angewandte Wissenschaften (ZHAW), Zurich, Switzerland; ^3^Institute for Computational Science, Universität Zürich (UZH), Zurich, Switzerland; ^4^Bruker Schweiz AG, Fällanden, Switzerland

**Keywords:** Nuclear Magnetic Resonance, automatic signal classification, deep learning, ^1^H spectra, multiplet assignment

## Abstract

The identification and characterization of signal regions in Nuclear Magnetic Resonance (NMR) spectra is a challenging but crucial phase in the analysis and determination of complex chemical compounds. Here, we present a novel supervised deep learning approach to perform automatic detection and classification of multiplets in ^1^H NMR spectra. Our deep neural network was trained on a large number of synthetic spectra, with complete control over the features represented in the samples. We show that our model can detect signal regions effectively and minimize classification errors between different types of resonance patterns. We demonstrate that the network generalizes remarkably well on real experimental ^1^H NMR spectra.

## Graphical Abstract

We present a novel supervised deep learning approach to perform automatic detection and classification of multiplets in ^1^H Nuclear Magnetic Resonance (NMR) spectra. 
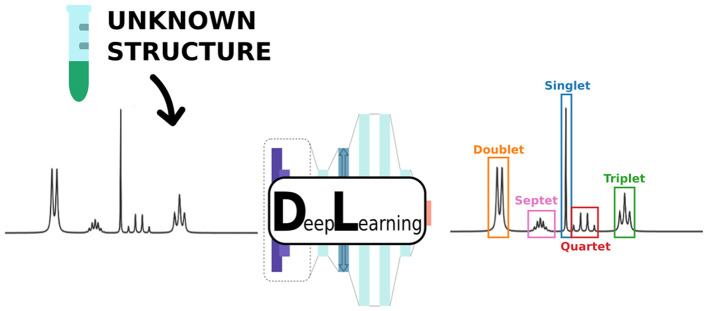

## 1. Introduction

Since its discovery, Nuclear Magnetic Resonance (NMR) spectroscopy has become an effective and reliable tool for investigating complex molecular compounds, using the interaction of nuclear spins, an intrinsic property of atoms, with the magnetic field. An NMR spectrum contains different kinds of resonances as a function of frequency, including isolated peaks, referred to as *singlets*, double peaks, referred to as *doublets*, up to composite sets of multiple peaks, generally referred to as *multiplets* (Keeler, 2002). The frequency coordinates of the multiplet (*chemical shift*), together with the integration of the multiplet profile, the resonance pattern and the distance between consecutive peaks within the same multiplet (*coupling constant*), serve as a *molecular fingerprint*. These features provide knowledge about the abundances of the atoms, their local chemical environments within the molecule and their connectivity and stereochemistry.

The traditional field in which NMR spectroscopy is extensively employed is organic chemistry, where it is used for the structure elucidation of new natural compounds and reaction products (Jackmann and Sternhell, 1969). Yet, NMR spectroscopy is widespread in numerous scientific fields, combining both qualitative and quantitative approaches. The information extracted from chemical shifts, coupling constants and peak integration is used for the study of the dynamics and compartmentation of metabolic pathways in system biochemistry (Fan and Lane, 2016), for the diagnosis of tumors, hematomas, and other pathologies (e.g., multiple sclerosis) in medicine (Zia et al., 2019), for characterization of humic substances and analysis of contaminants in environmental sciences (Cardoza et al., 2004), and for the evaluation of soil components, plant tissues and complex food compounds in agriculture (Mazzei and Piccolo, 2017) and food chemistry (Cao et al., 2021).

Nevertheless, there is a major drawback. The process of retrieving information from the spectra is often very demanding, time-consuming and susceptible to errors. It requires the involvement of expert spectroscopists to perform manual annotation of the spectra, chemical shift and coupling constants extraction, and structure elucidation. Moreover, the evaluation and interpretation of the NMR spectra are not always straightforward and unambiguous, due to the presence of spectral artifacts and overlapping resonances. Therefore, introducing automation in the NMR analysis could accelerate and facilitate the process while increasing the robustness and reproducibility of the results.

One of the tasks that can considerably benefit from the introduction of automation is the annotation of the signal regions for their coupling constants patterns. Early automated approaches were based on local symmetry properties (Boentges et al., 1989), maximum entropy techniques (Delsuc and Levy, 1988; Seddon et al., 1996; Stoven et al., 1997), multiplets deconvolution (Jeannerat and Bodenhausen, 1999) and pattern recognition (Golotvin and Chertkov, 1997). Hoye proposed a recursive algorithm (Hoye et al., 1994; Hoye and Zhao, 2002) to deduce the splitting tree of a multiplet which has successively been supplemented with symmetry and amplitude constraints (Golotvin et al., 2002; Cobas et al., 2005). These approaches have the limitation that they are meant to be applied to one resonance at a time and need prior information on the expected number of peaks involved. Griffiths (2000) introduced an automatic procedure to aggregate individual peaks into multiplets over the entire spectrum. Nonetheless, the model relies on the knowledge of the exact position of the peaks, which hinders the complete automation of the task. Even if classical methodologies are still employed (Jeannerat and Cobas, 2021), the attention has recently shifted toward Deep Learning for its unique power to reach human-level performance and above on image classification, speech recognition, and natural language processing (LeCun et al., 2015; Baraniuk et al., 2020). The potentiality of deep learning has been employed to perform different stages of the NMR analysis (Chen et al., 2020; Cobas, 2020), from reconstruction and denoising of the signal to the interpretation of the spectra, including chemical shift prediction (Jonas et al., 2022), automated peak picking and spectral deconvolution (Paruzzo et al., 2019; Li et al., 2021; Schmid et al., 2022), to the fully automated structure verification (Klukowski et al., 2022).

However, to the best of our knowledge, a deep learning based algorithm, able to recognize resonance patterns of multiplets over the entire one-dimensional spectrum without human intervention and without any prior information on the structure of the molecule, is still missing.

Here we introduce a supervised deep learning model that performs automated detection and classification of signal regions in one-dimensional NMR spectra. The network's architecture includes a combination of one-dimensional convolutional layers, Long Short Term Memory layers and fully connected layers, which has proven to be effective in sequences and signal analysis (Sainath et al., 2015; Mutegeki and Han, 2020; Xu et al., 2020; Tasdelen and Sen, 2021; Ozkok and Celik, 2022). Similarly to a manual annotation procedure, the output is a point-by-point prediction of a label value that corresponds to a given resonance pattern. The network is trained and quantitatively tested on synthetic spectra and on 10 experimental spectra of small molecules, yielding a highly accurate and robust method for the characterization of non-overlapping resonances in ^1^H NMR spectra.

## 2. Method

We implemented a supervised deep learning algorithm to detect and localize signal regions in ^1^H NMR spectra and classify them with respect to their resonance patterns into seven multiplet classes: singlets, doublets, triplets, quartets, quintets, sextets and septets. ^1^H NMR spectra are generally characterized by the presence of resonances partially or totally overlaying other resonances. These so-called *overlapping multiplets* were excluded from the present analysis. The prediction was based on the shape of the resonances only, without any prior information on the molecule giving rise to the spectrum and its structure.

### 2.1. Training set

The model was trained with 100,000 synthetic segments of one-dimensional NMR spectra. Generating the input data from scratch has various advantages. Deep learning algorithms are known to require a large number of samples to be trained on, and artificial production ensures a virtually unlimited availability of samples. The labeling of the spectra can be carried out automatically when creating the spectra. This overcomes the difficulties of finding an extensive dataset annotated by NMR experts with a costless, fast and robust procedure. Moreover, generating the samples guarantees total control over the features represented, making it possible to tailor the training set toward the demands of the task.

The segments of the spectra were all simulated with 1,024 points over a range of 0.512 ppm with a base frequency of 400 MHz. The number of signal regions included in a single spectrum segment decays exponentially. The resonance patterns to be included in each spectrum were sampled randomly between seven multiplet classes: singlets, doublets, triplets, quartets, quintets, sextets and septets. In NMR theory, the peaks have a Lorentzian profile (Keeler, 2002). However, due to imperfect experimental conditions, the peak profile results in a convolution of the Lorentzian lineshape with a Gaussian lineshape, the Voigt profile, which can be reproduced with the following *Pseudo-Voigt approximation* (Kielkopf, 1973):


(1) $$\begin{array}{l} V\left(\omega -\omega_{0};\gamma ,\sigma \right)\equiv l_{s}G\left(\omega -\omega_{0};\sigma \right)+\left(1-l_{s}\right)L\left(\omega -\omega_{0};\gamma \right) \end{array}$$

Where ω_0_ is the maximum position, *G* is the Gaussian lineshape and σ is its variance, while *L* is the Lorentzian lineshape and γ is its Half Width at Half Maximum (HWHM). Gaussian and Lorentzian lineshapes were generated with the same HWHM, so that $\gamma \equiv \sigma \sqrt{2\log\left(2\right)}$. The parameter γ was sampled between 0.5 and 7*Hz*, while the parameter *l*_*s*_ was sampled between 0, which corresponds to the Lorentzian limit, and 1, which corresponds to the Gaussian limit. The amplitude of the multiplets was sampled to reproduce the high dynamic range usually found in experimental spectra. The ratio of the distance between consecutive peaks in a multiplet, which for the resonance patterns considered corresponds to the coupling constant *J*, and the Full Width at Half Maximum (FWHM) of the peaks 2γ, was varied between 0.5 and 18 to achieve different levels of signal resolution. In this context a multiplet with a higher resolution has well separated peaks. To make the synthetic spectra as similar as possible to their experimental counterpart, different kinds of effects and distortions were introduced. A given amount of Gaussian noise was added to the samples so as to have a signal to noise ratio between 10^1/2^ and 10^5/2^. Moreover, small phase and baseline distortions were introduced (see Supplementary material), in order to make the algorithm's prediction more robust over experimental input spectra which show residual phase and baseline distortions after correction. Finally, to mimic the *rooftop effect* (Keeler, 2002) due to strong coupling interactions, we slightly altered the theoretically expected amplitude ratios of the peaks.

Once the multiplets were generated, the spectra were labeled, assigning to each point the value of the class it belongs to. The multiplet label set is a subset of the Natural numbers C≡{0,1,2,3,4,5,6,7}⊂ℕ. The numbers from 1 to 7 were used for singlets to septets classes, while the baseline points were marked with the number 0.

### 2.2. Network architecture

**Figure 1:**
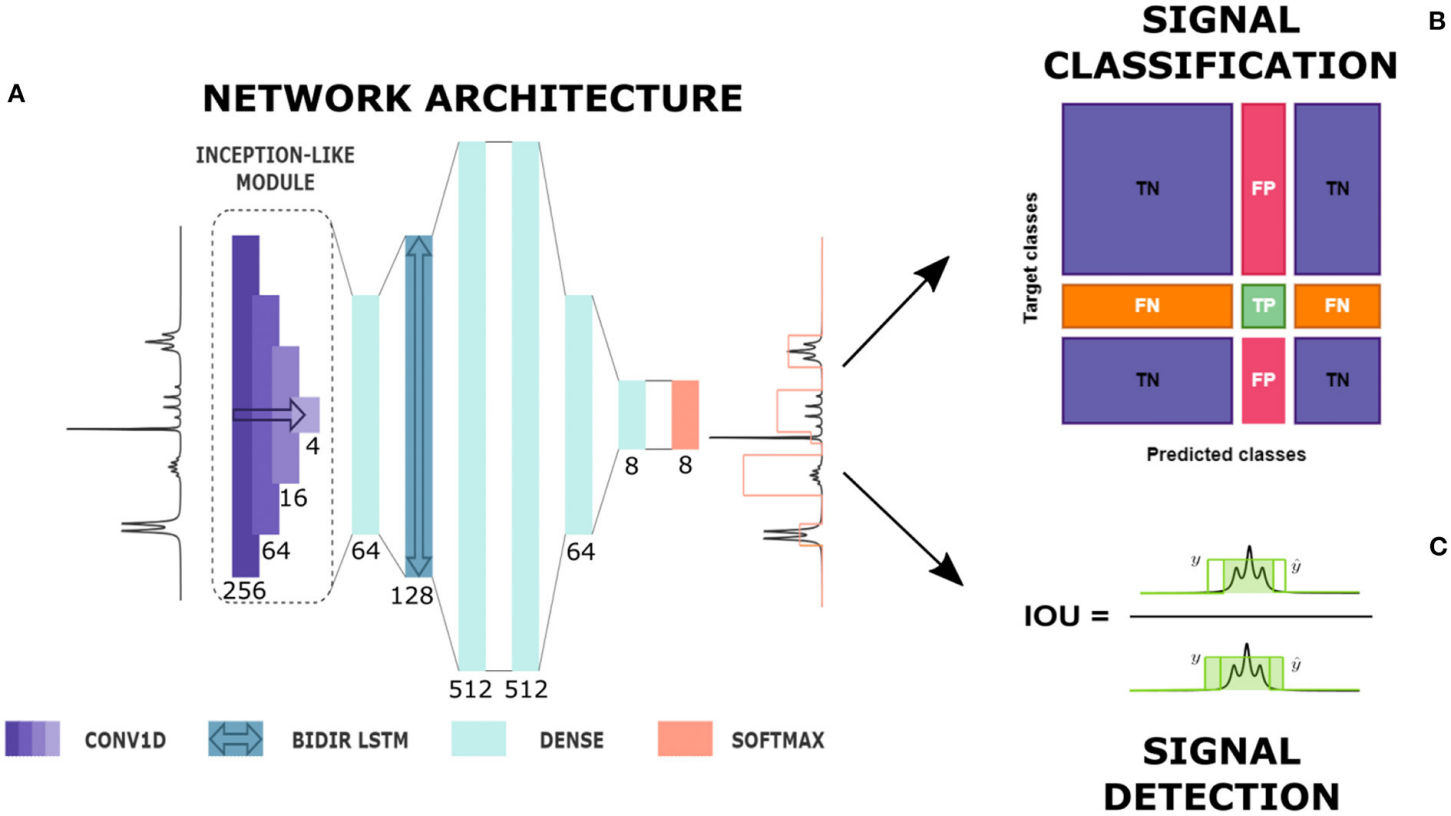
From automatic prediction to analysis. **(A)** Diagram of network's architecture. CONV1D is a one-dimensional convolutional layer, BIDIR LSTM is a bidirectional LSTM layer, DENSE is a time-distributed fully connected layer and SOFTMAX is the softmax output. Here, the type of each layer with its kernel size, in the case of convolutional layers, and number of units, in the case of fully connected and LSTM layers are given (see also Supplementary material). The network produces the prediction of a label whose height represents the multiplet class. **(B)** Signal classification. True Positives (TP), False Positives (FP), True Negatives (TN) and False Negatives (FN) definition for a given multiplet class based on the confusion matrix entries. **(C)** Signal detection: Intersection Over Union (IOU) definition in a 1D framework.

The network implemented for the detection and classification of multiplets was characterized by an architecture similar to the one used by Schmid et al. (2022) for the spectral deconvolution task. The NMR spectrum was first sent to an Inception-like module (Szegedy et al., 2015) composed of four 1D convolutional layers of 16 filters with kernel sizes of 4, 16, 64, and 256 (see Figure 1). Each layer was followed by an Exponential Linear Unit (ELU) activation function. The range covered by a signal region varies with the line width and the number of peaks included in that region (e.g., a singlet is usually less extended than a septet). Including an Inception-like module assures the possibility of reaching higher levels of complexity, extracting features at different length scales while reducing computational expense and mitigating overfitting on the training set. After a time-distributed fully connected layer, the spectrum passed through a Bi-directional Long Short Term Memory (LSTM) layer. Bi-directionality means that information flows in both directions across the repeating modules of the layer. This component was decisive in our case to ensure that the prediction of each spectrum point was determined by the features appearing on both of its sides and that the positioning of the multiplets along the frequency axis did not affect the classification.

The successive layers were four time-distributed dense layers with decreasing number of nodes, from 512 to the number of classes, followed by a Rectified Linear Unit (ReLU) activation function. In the end, a softmax output produced for each point in the spectrum the probability of belonging to every multiplet class considered.

During training, the network's architecture received a series of synthetic input samples ***x*** with their corresponding labels ***y***. All the 1, 225, 606 trainable weights θ were updated at each learning epoch in order to minimize the Categorical Cross-Entropy loss function. The network was trained for 33 epochs on a HP Z2 Tower G5 Workstation with Intel(R) Core(TM) i7-10700 CPU and CometLake-S GT2 [UHD Graphics 630] GPU. To track the training process we split this set into two subgroups. The first subgroup was composed of 75, 000 segments meant for the training, the second subgroup was composed of 25, 000 segments meant for validation. At the end of the training phase, the network had learnt a complex function of the inputs Φ_θ_(***x***) and, considering the most probable multiplet class over all classes *l*, was able to output the label prediction vector $\hat{y}=\underset{l}{\max}\text{Softmax}\left(l\right)$. When running the model over entire experimental spectra, the network's input was resized in order to match the number of spectral points. To improve the prediction, the experimental spectra were resampled so as to obtain the same number of points per *Hz* as in the training set.

### 2.3. Evaluation metrics for classification

Since the network produces a prediction point-by-point, the most straightforward approach to evaluating the network's performance was to check if each point in the spectra was correctly classified within the different multiplet classes. We refer to this approach as the *point-wise approach*. However, a considerable concern is that of assessing the prediction, not just on the individual points, but on the multiplet as a whole. We refer to this second approach as the *object-wise approach*.

#### 2.3.1. Point-wise approach

We calculated the *confusion matrix* for the multi-class classification problem of discriminating among various types of multiplets (Padilla et al., 2020). A confusion matrix is a square cross table **C** where the columns represent the predicted classes and the rows represent the actual classes. Each element of the matrix *C*_*ij*_ contains the number of points belonging to class *i*, which are predicted by the network within class *j*. From the confusion matrix, four performance indicators can be defined, True Positives (TP), False Positives (FP), False Negatives (FN) and True Negatives (TN), as displayed in Figure 1. These indicators help to interpret the specific nature of the errors the network produces, pointing out the sources of confusion for the classification algorithm. For each multiplet class, we measured (Hossin and Sulaiman, 2015) *accuracy* (A), which is the fraction of properly classified points in the spectra, *precision* (P), which is the rate of correct predictions in a given class over all the predictions in that class, *recall* (R), which is the rate of correct predictions among all ground truths in a given class, and *F1 score*, which is the harmonic average of precision and recall. An efficient classifier should maximize both precision and recall, that is minimize false positives and false negatives at the same time. Therefore, the F1 score is a reliable indicator of the network's performance because it represents a midpoint between precision and recall.

#### 2.3.2. Object-wise approach

To evaluate the classification model on whole signal regions, we computed the *precision-recall curve*, adapting the method in Padilla et al. (2020) to our one-dimensional case. We defined a *label prediction* L as the connected set of points marked with a given label *l*, $L\equiv \left\{i\in \text{spectrum points}|{\hat{y}}_{i}=l\right\}$, and surrounded either by baseline points or points marked by other labels. We defined the *confidence* of each label prediction as the average of the softmax output of the points belonging to that label prediction:


(2) $$\begin{array}{l} \text{Confidence}\left(L\right)=\frac{1}{n_{points}}\overset{n_{points}}{\underset{i\in L}{\sum }}Softmax_{i}\left(l\right). \end{array}$$

All predicted labels, except for the baseline label, were ranked with their confidence in decreasing order. For each prediction, the Intersection Over Union (IOU) metrics was computed. As the name says, IOU is the ratio of the intersection between the area of the predicted label and the area of the ground truth label, over the union of those areas. In our one-dimensional case where the ground truth vector is ***y*** and the predicted vector is $\hat{y}$, the IOU becomes:


(3) $$\begin{array}{l} \text{IOU}=\frac{\hat{y}\cap y}{\hat{y}\cup y}. \end{array}$$

Going down the ranked list of label predictions, each prediction was considered a true positive if its IOU was above a threshold *t*, which is usually set to 0.50 or 0.75. Otherwise, it was considered a false positive. Then, for each prediction in the list, we computed precision as a function of increasing recall, obtaining a precision-recall curve. Precision was measured as the ratio between the true positives encountered so far in the list over all the predictions encountered so far, while recall was measured as the ratio between the true positives encountered so far in the list over the total number of predictions.

The Area Under the Curve (AUC) of the precision-recall curve, often referred to as Average Precision (AP), was measured with an interpolation procedure over the points *n* of the curve:


(4) $$\begin{array}{l} \text{AP}=\underset{n}{\sum }\left(R_{n+1}-R_{n}\right)P_{\text{interp}}\left(R_{n+1}\right) \end{array}$$

with


(5) $$\begin{array}{l} P_{\text{interp}}\left(R_{n+1}\right)=\underset{\tilde{R}\geq R_{n+1}}{\max}P\left(\tilde{R}\right), \end{array}$$

where *P* and *R* denote, respectively, the precision and recall values.

Obtaining a good result for the object-wise statistics not only ensures that a large fraction of spectral points is correctly classified, as can already be demonstrated through point-wise statistics, but it also ensures that the prediction is not fragmented. A fragmented prediction occurs when different sets of spectral points belonging to the same signal region are classified within different multiplet classes. This is a common issue for algorithms that produce a point-by-point output. When inside a signal region there are multiple class predictions, each connected set is considered as an individual label prediction with a modest intersection with the ground truth region, leading to a decline in the performance.

## 3. Results

### 3.1. Synthetic spectra

**Figure 2:**
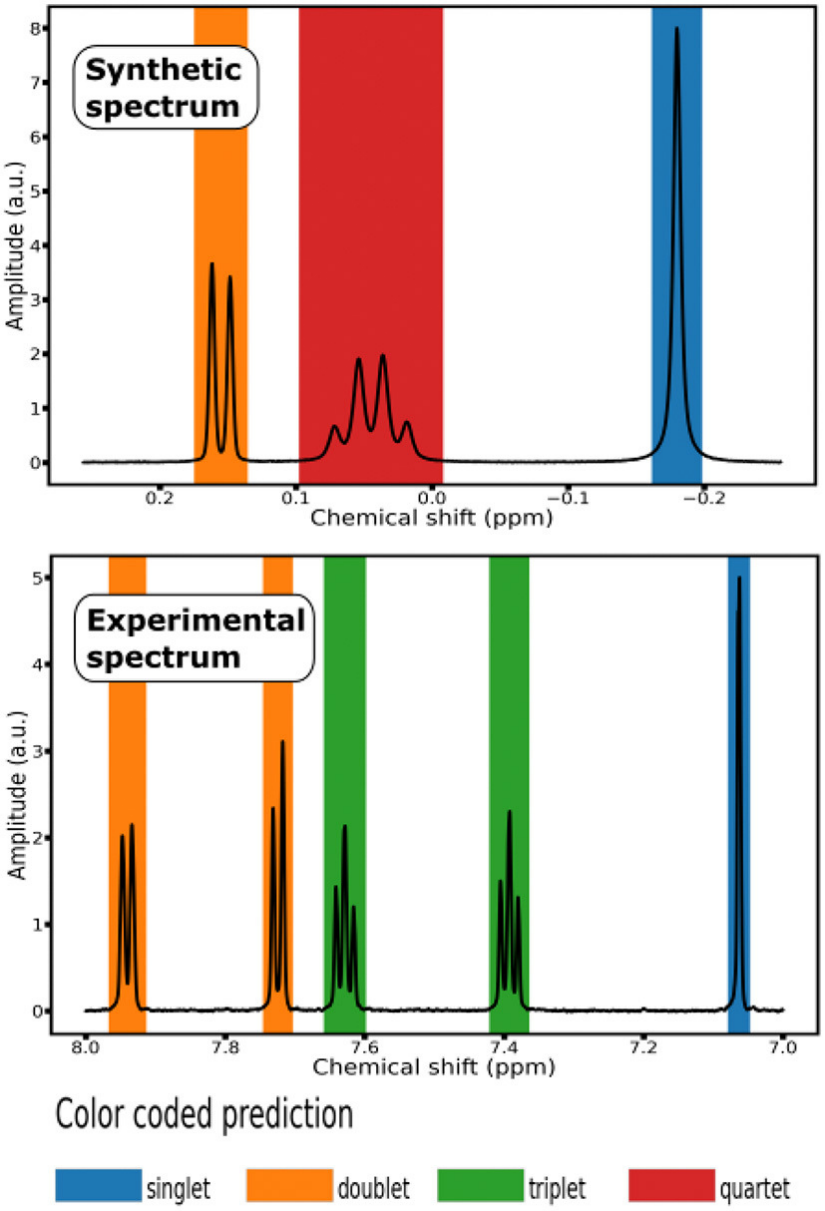
Color coded prediction of ^1^H NMR Spectra: **(top)** segment of a synthetic spectrum; **(bottom)** segment of an experimental spectrum from the testing set of 10 small molecules.

**Figure 3:**
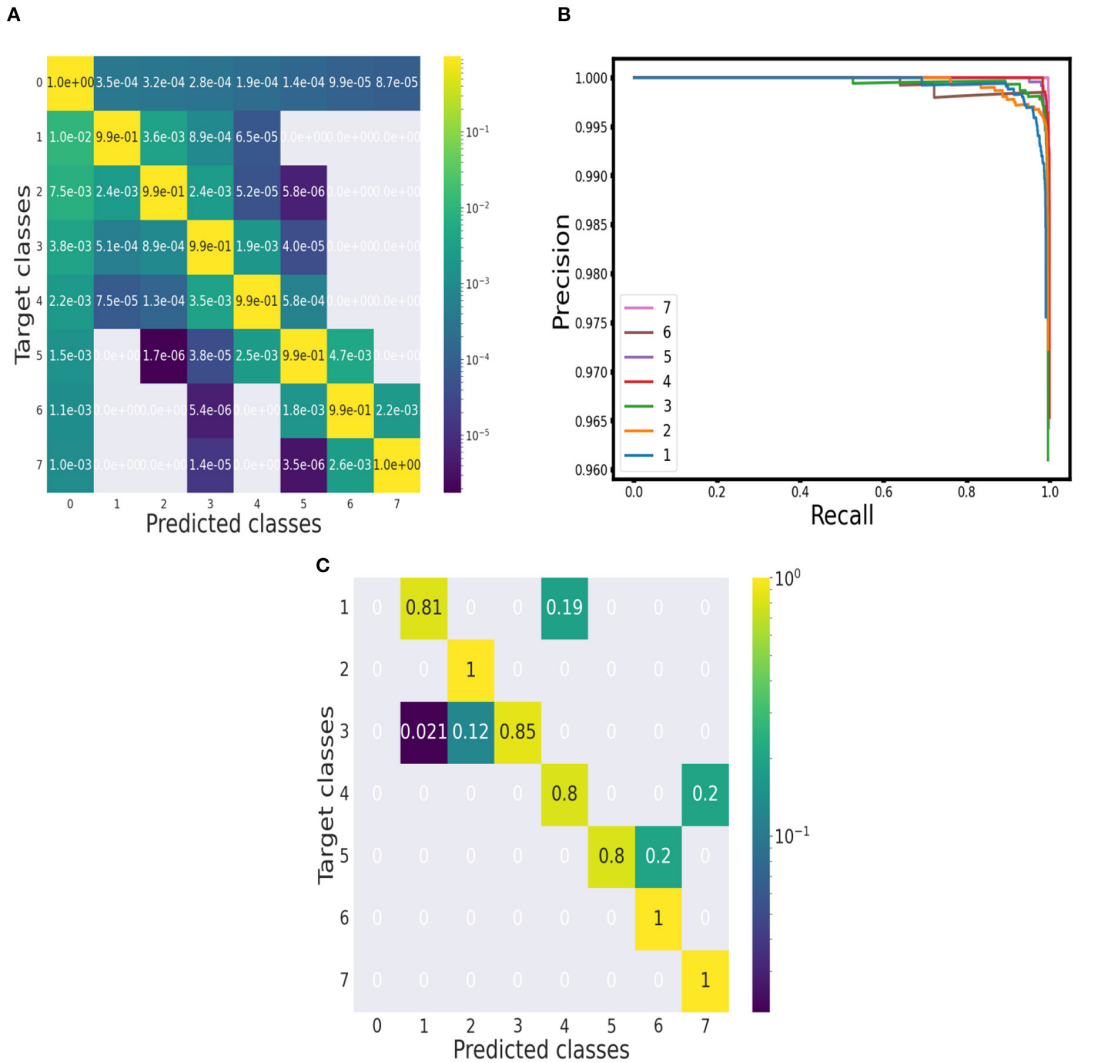
Evaluation of performance. **(A)** Point-wise approach: Confusion matrix of the performance on synthetic spectra. **(B)** Object-wise approach: Precision-recall curves for each multiplet class (baseline class excluded) at threshold 75%. **(C)** Point-wise approach: Confusion matrix of the performance on the 10 ^1^H NMR spectra of the testing set.

We run the classification model over 10,000 segments of synthetic spectra generated independently from the training set. An example of the classification over a synthetically produced segment is displayed in Figure 2. The performance of the prediction for each multiplet class is reported in the confusion matrix in Figure 3. The entries of the matrix were normalized along the rows, that is over the total number of ground truth points in each class. The true positive rates on the diagonal of the matrix are all above 99%. Even if with a very low rate, the most frequent errors involve the baseline class: either baseline points are predicted as signal points or signal points are predicted as baseline points. After a visual inspection, it was apparent that these errors happen at the borders of a signal region and can be explained with a slightly different positioning, of the order of a few points, of the predicted label with respect to the ground truth one. However, these positioning inaccuracies of the predicted labels are negligible compared to a serious misinterpretation of a noise region for signal. Considering the error rates of the multiplet classes, it appears that as the number of peaks in the multiplet class increases the error rate decreases, with the highest error rate, of the order of 1.5%, belonging to the singlets class. This behavior can be interpreted considering the presence of a slight *class imbalance*. In a point-by-point classification algorithm, this issue exists despite the adoption of a synthetic training set. When generating the training set, the pattern of the resonances is chosen randomly so that there will be on average the same number of resonances for each multiplet class. However, the extension of multiplets varies with the number of peaks so that resonances with more peaks spread over a larger number of spectral points. Therefore, during training, the network is presented with more points belonging to multiplets with a higher number of peaks. This behavior should be considered attentively. However, the excellent results achieved even in the case of singlets assure the proper functioning of the classification algorithm.

From the confusion matrix, we measured accuracy, precision, recall and F1 score. The results for each multiplet class together with an average over all the classes are reported in Table 1. Accuracy is always above 99% and precision, recall and F1 score do not fall below 98.5%. Precision and recall values do not diverge significantly across the multiplet classes, with a difference of 0.21% on average. Therefore, the model is able to successfully minimize false positives and false negatives at the same time. This can be confirmed by analyzing the precision-recall curves (see Figure 3). An optimal classification algorithm would yield a precision of 1.0 for all values of recall. Our model is approaching this limit. The choice of the IOU threshold whereby a label prediction was identified as a true positive or a false positive is arbitrary and, indeed, each value of the IOU threshold defines a different AP metric. In the present work, we reported for each class the AP metric and the average over all classes (mAP) for two threshold values, 50% and 75% (see Table 1). Increasing the IOU threshold increases the probability that a label prediction is a false positive and deteriorates the overall performance metrics. On average, passing from a threshold of 50% to a threshold of 75% decreases the AP metrics by only 0.47%. Also in the contest of the object-wise approach, it is shown that the multiplet class for which the classification efficiency declines faster when increasing the IOU threshold is that of singlets.

**Table 1:** Point-wise approach: Accuracy, precision, recall and F1 score metrics are reported for all the multiplet classes; the last row shows the average performance over the classes.

	**A(%)**	**P(%)**	**R(%)**	**F1(%)**	**AP50(%)**	**AP75(%)**
Class 0	99.8	99.9	99.9	99.9	—	—
Class 1	99.9	98.6	98.5	98.6	100	98.9
Class 2	99.9	99.0	98.8	98.9	100	99.5
Class 3	99.9	99.0	99.3	99.1	100	99.5
Class 4	99.9	99.3	99.4	99.3	100	99.8
Class 5	99.9	99.6	99.1	99.4	99.8	99.4
Class 6	99.9	99.1	99.5	99.3	100	99.5
Class 7	100	99.7	99.6	99.7	99.8	99.7
Average	99.9	99.3	99.3	99.3	100	99.5

Object-wise approach: The Area Under the Curve (AUC) of precision-recall curves, the Average Precision (AP), at threshold 50% and 75% is reported for all multiplet classes (baseline class excluded); the last row shows the mean Average Precision (mAP).

### 3.2. Experimental spectra

When training a deep neural network with a set of synthetic data, a robust generalization toward real data, that is experimental ^1^H NMR spectra, becomes a crucial and non-trivial objective to meet. Supervised learning models generally assume that the input samples in the training set are drawn from the same probability distribution as the samples that will be fed into the network during testing: *P*_train_(***y***|***x***) = *P*_test_(***y***|***x***), where ***x*** is the input sample vector, and ***y*** is the label vector. However, when applying the classification algorithm over experimental spectra, their distribution could deviate, even significantly, from that of the synthetic spectra in the training set: *P*_train_(***y***|***x***)≠*P*_test_(***y***|***x***). When the hypothesis of having no change between training and testing samples distribution fails, we are dealing with a *distribution shift* (Quiñonero-Candela et al., 2009) which may cause a deterioration of the prediction's accuracy. Recent deep learning applications toward spectral denoising (Lee and Kim, 2019), spectral reconstruction of Non-Uniformely Sampled (NUS) datasets (Hansen, 2019; Qu et al., 2020), peak picking and spectral deconvolution (Li et al., 2021; Schmid et al., 2022) have proven that it is feasible to obtain outstanding results on NMR experimental data with networks trained on synthetic data.

To evaluate the generalization properties of our classification model, we run it on a testing set composed of experimental ^1^H NMR spectra of 10 *small molecules*, that is organic compounds with a low molecular weight ( ≤ 1, 000 daltons). Each experimental spectrum contains either 32, 768 or 65, 536 points (see Supplementary Table S3). To display the prediction of the labels, we associated each multiplet class with a color and shaded the signal region with the color corresponding to the predicted class (see Figure 2).

The experimental testing set was annotated by NMR experts, who labeled each peak with its features: position in ppm, amplitude, line width, line shape, and the multiplet class it belongs to.

To evaluate the classification performance of the model over the experimental set quantitatively, we built a confusion matrix (see Figure 3), considering each annotated peak and checking if its position fell into a spectral region marked with the label of the correct multiplet class. All the doublets, sextets and septets appearing in the testing set were properly classified and the true positive rates for the other classes were always above 80%.

## 4. Discussion

In this paper, we have presented a supervised deep learning network able to mimic the work of an expert spectroscopist who annotates one-dimensional NMR spectra produced by small molecules to retrieve information on their structure. One-dimensional NMR spectra have the most straightforward measurement design among the NMR experiments. Compared to advanced multi-dimensional techniques, which have acquisition times ranging from a few hours to days, one-dimensional spectra can be collected in a few minutes, leading to a drastic reduction in experimental costs. All these reasons make one-dimensional experiments the preferable methodology for the rapid evaluation of chemical compounds. Unfortunately, one-dimensional spectra are also the least informative measure. For example, the analysis of cross-peaks in two-dimensional NMR spectra, where each point has two frequency coordinates, easily leads to the identification of pairs of spins that couple to one another, significantly facilitating the structure elucidation process (Klukowski et al., 2022). In ^1^H NMR spectra, on the other hand, this same information can only be retrieved by evaluating at the same time the chemical shift and the pattern of the resonances. Considering these limitations, all classical approaches to the automation of one-dimensional NMR analysis are usually performed on individual multiplets (Golotvin et al., 2002; Cobas et al., 2005; Jeannerat and Cobas, 2021), which implies a previous knowledge of the precise location of the signal regions, and needs additional information on the number or positions of the peaks (Griffiths, 2000). Our algorithm detects and classifies multiplets over the entire spectrum simultaneously. This leads to a significant improvement since the network produces a multiplet prediction considering a larger portion of the spectrum than just the single signal region and extracting information also from neighboring resonances.

Essentially, the network performs a visual inspection of the spectrum, extracting features at different length scales through the application of convolutional layers of diverse kernel sizes. Then, the LSTM layer learns the correlations along the frequency axis and the fully connected layers map the extracted features into a more separable space, to help the classification. The problem of locating and identifying resonance patterns in NMR spectra may be compared to image recognition and object detection tasks. Conceivably, the same function can be exploited by one-dimensional adaptations of popular object detection network's architectures (He et al., 2016; Redmon et al., 2016; Ren et al., 2017). However, the effective combination of convolutional, LSTM and fully connected layers has already proven to be especially suitable to analyze and classify one-dimensional data, such as sequences and signals collected through diverse measurements, extracting features and then correlating them over the data points (Sainath et al., 2015; Mutegeki and Han, 2020; Xu et al., 2020; Tasdelen and Sen, 2021; Ozkok and Celik, 2022). Moreover, this kind of architecture allows keeping the number of layers reduced compared to object-detection architectures, achieving robust results at a lower computational cost.

When predicting the multiplet class point-by-point along the spectrum, a common issue that can be encountered is a fragmented classification over the signal region, especially when facing uncertain cases. This can be easily overcome by applying a majority filter as a post-processing step.

The generation of the training set was a key aspect of the realization of our model. Synthetic production of spectra assured a virtually unlimited availability of samples with a high level of diversity, paired with an automatic, costless and robust labeling procedure. As we pointed out in the Results section, when using a point-wise inducer class imbalance cannot be eradicated completely even with a synthetic tailored training set, because multiplet classes with a higher number of peaks spread over a larger set of spectral points. Considering the excellent results achieved in all multiplet classes, the presence of a slight class imbalance did not hinder the overall classification performance. Nonetheless, the different amounts of points for different classes should be taken into account carefully at implementation time, adding patterns of underrepresented classes or pruning patterns of overrepresented ones if needed.

Besides, multiplets generated synthetically should closely mirror the shapes and resonance patterns encountered in real experimental spectra, to mitigate the effect of distribution shift on the generalization performance. In this respect, complex resonance patterns not represented in the training set cannot be recognized and classified properly at the present stage. A future study should include multiplet classes characterized by several coupling splittings. Encountering these kinds of resonances in ^1^H NMR spectra is becoming more and more frequent with the increasing measurement resolution reached by the new benchtop spectrometers.

Even with a higher resolution, one-dimensional spectra are usually marked by an extensive presence of resonances laying over other resonances: the so-called *overlapping multiplets*. Hence, the motivation for restricting the usage of one-dimensional NMR spectra to the analysis of small molecules. Macromolecules, such as proteins, including a much larger number of atoms, will produce excessively convoluted overlapping resonances which might lead to absolutely inconclusive results for the analysis.

Attempting to classify overlapping peaks is a challenging task for a neural network: the signal of several resonances can add up in countless fashions, making it difficult to define specific features for such a class. One possibility could be to build the overlapping class with every resonance that cannot fit into any other multiplet class. We plan to investigate this in a forthcoming work.

## 5. Conclusion

In conclusion, the proposed deep learning framework has demonstrated the ability to effectively detect and classify signal regions in ^1^H NMR spectra. The network has produced outstanding results when applied to synthetic spectra, reaching an average of 99.9% for accuracy metrics and 99.3% for precision, recall and F1 score. Moreover, the learning algorithm has proven to be capable of generalizing remarkably well on real experimental ^1^H NMR spectra. Providing scientists with an efficient and reliable tool to discriminate the various classes of resonances in one-dimensional spectra, without the need of turning to more sophisticated and expensive techniques, holds the promise of speeding up the workflow of production and analysis of chemical compounds while introducing a higher level of consistency among experimental results.

## Data availability statement

The original contributions presented in the study are included in the article/Supplementary material, further inquiries can be directed to the corresponding author.

## Author contributions

GF developed the code, performed the simulations, and produced the results. NS contributed to the development of the method. SB contributed to the generation of the data set, development of the method, and discussion of the results. AH and DW contributed to the review and discussion of the results. AS and GC contributed to the discussion of the results. All authors contributed to the manuscript revision and read and approved the submitted version.

## References

[B1] Baraniuk R. Donoho D. Gavish M. (2020). The science of deep learning. Proc. Natl. Acad. Sci. U.S.A. 117, 30029–30032. 10.1073/pnas.2020596117 33229565 PMC7720210

[B2] Boentges S. Meier B. Griesinger C. Ernst R. (1989). Local symmetry in 2D and 3D NMR spectra. J. Magn. Reson. 85, 337–358. 10.1016/0022-2364(89)90148-012522306

[B3] Cao R. Liu X. Liu Y. Zhai X. Cao T. Wang A.. (2021). Applications of nuclear magnetic resonance spectroscopy to the evaluation of complex food constituents. Food Chem. 342, 1–10. 10.1016/j.foodchem.2020.128258 33508899

[B4] Cardoza L. Korir A. Otto W. Wurrey C. Larive C. (2004). Applications of NMR spectroscopy in environmental science. Prog. Nucl. Magn. Reson. Spectrosc. 45, 209–238. 10.1016/j.pnmrs.2004.06.00233130521

[B5] Chen D. Wang Z. Guo D. Orekhov V. Qu X. (2020). Review and prospect: deep learning in nuclear magnetic resonance spectroscopy. Chem. Eur. J. 26, 10391–10401. 10.1002/chem.202000246 32251549

[B6] Cobas C. (2020). NMR signal processing, prediction, and structure verification with machine learning techniques. Magn. Reson. Chem. 58, 512–519. 10.1002/mrc.4989 31912547

[B7] Cobas J. Constantino-Castillo V. Martìn-Pastor M. del Rìo-Portilla F. (2005). A two-stage approach to automatic determination of 1H NMR coupling constants. Magn. Reson. Chem. 43, 843–848. 10.1002/mrc.1623 16025552

[B8] Delsuc M. Levy G. (1988). The application of maximum entropy processing to the deconvolution of coupling patterns in NMR. J. Magn. Reson. 76, 306–315. 10.1016/0022-2364(88)90112-6

[B9] Fan T. Lane A. (2016). Applications of NMR spectroscopy to systems biochemistry. Prog. Nucl. Magn. Reson. Spectrosc. 92–93, 18–53. 10.1016/j.pnmrs.2016.01.005 26952191 PMC4850081

[B10] Golotvin S. Chertkov V. (1997). Pattern recognition of the multiplet structure of NMR spectra. Russian Chem. Bull. 46, 423–430. 10.1007/BF02495389

[B11] Golotvin S. Vodopianov E. Williams A. (2002). A new approach to automated first-order multiplet analysis. Magn. Reson. Chem. 40, 331–336. 10.1002/mrc.1014

[B12] Griffiths L. (2000). Towards the automatic analysis of 1H NMR spectra. Magn. Reson. Chem. 38, 444–451. 10.1002/1097-458X(200006)38:6andlt;444::AID-MRC673andgt;3.0.CO;2-Z 15390025

[B13] Hansen D. (2019). Using deep neural networks to reconstruct non-uniformly sampled NMR spectra. J. Biomol. NMR. 73, 577–585. 10.1007/s10858-019-00265-1 31292846 PMC6859195

[B14] He K. Zhang X. Ren S. Sun J. (2016). “Deep residual learning for image recognition,” in 2016 IEEE Conference on Computer Vision and Pattern Recognition (CVPR) (Las Vegas, NV: IEEE), 770–778.

[B15] Hossin M. Sulaiman M. (2015). A review on evaluation metrics for data classification evaluations. Int. J. Data Min. Knowl. Manag. Process 5, 1–11. 10.5121/ijdkp.2015.5201

[B16] Hoye T. Hanson P. Vyvyan J. (1994). A practical guide to first-order multiplet analysis in 1H NMR spectroscopy. J. Org. Chem. 59, 4096–4103. 10.1021/jo00094a018 12054933

[B17] Hoye T. Zhao H. (2002). A method for easily determining coupling constant values: an addendum to “a practical guide to first-order multiplet analysis in 1H NMR spectroscopy”. J. Org. Chem. 67, 4014–4016. 10.1021/jo001139v 12054933

[B18] Jackmann L. Sternhell S. (1969). Applications of Nuclear Magnetic Resonance Spectroscopy in Organic Chemistry. New York, NY: Pergamon Press.

[B19] Jeannerat D. Bodenhausen G. (1999). Determination of coupling constants by deconvolution of multiplets in NMR. J. Magn. Reson. 141, 133–140. 10.1006/jmre.1999.1845 10527750

[B20] Jeannerat D. Cobas C. (2021). Application of multiplet structure deconvolution to extract scalar coupling constants from 1D nuclear magnetic resonance spectra. Magn. Reson. 2, 545–555. 10.5194/mr-2-545-2021 PMC10539725 37905213

[B21] Jonas E. Kuhn S. Schlörer N. (2022). Prediction of chemical shift in NMR: a review. Magn. Reson. Chem. 60, 1021–1031. 10.1002/mrc.5234 34787335

[B22] Keeler J. (2002). Understanding NMR Spectroscopy, New York, NY: Wiley.

[B23] Kielkopf J. (1973). New approximation to the Voigt function with applications to spectral-line profile analysis. J. Opt. Soc. Am. 63, 987–995. 10.1364/JOSA.63.000987

[B24] Klukowski P. Riek R. Güntert P. (2022). Rapid protein assignments and structures from raw NMR spectra with the deep learning technique ARTINA. Nat. Commun. 13, 2041–1723. 10.1038/s41467-022-33879-5 36257955 PMC9579175

[B25] LeCun Y. Bengio Y. Hinton G. (2015). Deep learning. Nature 521, 436–444. 10.1038/nature14539 26017442

[B26] Lee H. Kim H. (2019). Intact metabolite spectrum mining by deep learning in proton magnetic resonance spectroscopy of the brain. Magn. Reson. Med. 82, 33–48. 10.1002/mrm.27727 30860291

[B27] Li D. Hansen A. Yuan C. Brüschweiler-Li L. Brüschweiler R. (2021). DEEP picker is a deep neural network for accurate deconvolution of complex two-dimensional NMR spectra. Nat. Commun. 12, 5229–5242. 10.1038/s41467-021-25496-5 34471142 PMC8410766

[B28] Mazzei P. Piccolo A. (2017). HRMAS NMR spectroscopy applications in agriculture. Chem. Biol. Technol. Agric. 4, 2196–5641. 10.1186/s40538-017-0093-9

[B29] Mutegeki R. Han D. (2020). “A CNN-LSTM approach to human activity recognition,” in 2020 International Conference on Artificial Intelligence in Information and Communication (ICAIIC) (Fukuoka: IEEE), 362–366.

[B30] Ozkok F. Celik M. (2022). A hybrid CNN-LSTM model for high resolution melting curve classification. Biomed. Signal Process. Control. 71, 103168. 10.1016/j.bspc.2021.103168

[B31] Padilla R. Netto S. da Silva E. (2020). “A survey on performance metrics for object-detection algorithms,” in International Conference on Systems, Signals and Image Processing (IWSSIP) (Niteroi: IEEE), 237–242.

[B32] Paruzzo F. Bruderer S. Janjar Y. Heitmann B. Bolliger C. (2019). “Automatic signal region detection in ^1^H NMR spectra using deep learning,” in Bruker Whitepaper. p. 1–5.

[B33] Qu X. Huang Y. Lu H. Qui T. Guo D. Agback T.. (2020). Accelerated nuclear magnetic resonance spectroscopy with deep learning. Angewandte Chem. Int. Edn. 59, 10297–10300. 10.1002/anie.201908162 31490596

[B34] Quiñonero-Candela J. Sugiyama M. Schwaighofer A. Lawrence N. D. (2009). Dataset Shift in Machine Learning. Cambridge, MA: MIT Press.

[B35] Redmon J. Divvala S. Girshick R. Farhadi A. (2016). “You only look once: Unified, real-time object detection,” in 2016 IEEE Conference on Computer Vision and Pattern Recognition (CVPR) (Las Vegas, NV: IEEE), 779–788.

[B36] Ren S. He K. Girshick R. Sun J. (2017). Faster R-CNN: towards real-time object detection with region proposal networks. IEEE Trans. Pattern Anal. Mach. Intell. 39, 1137–1149. 10.1109/TPAMI.2016.2577031 27295650

[B37] Sainath T. Vinyals O. Senoir A. Sak H. (2015). “Convolutional, long short-term memory, fully connected deep neural networks,” in 2015 IEEE International Conference on Acoustics, Speech and Signal Processing (ICASSP) (South Brisbane, QLD: IEEE), 4580–4584.

[B38] Schmid N. Bruderer S. Paruzzo F. Fischetti G. Toscano G. Graf D.. (2022). Deconvolution of 1D NMR spectra: a deep learning-based approach. J. Magn. Reson. 347, 107357. 10.1016/j.jmr.2022.107357 36563418

[B39] Seddon M. Ferrige A. Sanderson P. Lindon J. (1996). Automatic recognition of multiplet patterns and measurement of coupling constants in NMR and EPR spectra through the application of maximum-entropy deconvolution. J. Magn. Reson. 119, 191–196. 10.1006/jmra.1996.0072

[B40] Stoven V. Annereau J. Delsuc M. Lallemand J. (1997). A new n-channel maximum entropy method in NMR for automatic reconstruction of “decoupled spectra” and J-coupling determination. J. Chem. Inf. Comput. Sci. 37, 265–272. 10.1021/ci960321n

[B41] Szegedy C. Liu W. Jia Y. Sermanet P. Reed S. Anguelov D.. (2015). “Going deeper with convolutions,” in Conference on Computer Vision and Pattern Recognition (CVPR) (Boston, MA: IEEE), 1–9.

[B42] Tasdelen A. Sen B. (2021). A hybrid CNN-LSTM model for pre-miRNA classification. Sci. Rep. 11, 14125. 10.1038/s41598-021-93656-0 34239004 PMC8266811

[B43] Xu G. Ren T. Chen Y. Che W. (2020). A one-dimensional CNN-LSTM model for epileptic seizure recognition using EEG signal analysis. Front. Neurosci. 14, 578126. 10.3389/fnins.2020.578126 33390878 PMC7772824

[B44] Zia K. Siddiqui T. Ali S. Farooq I. Zafar M. S. Khurshid Z. (2019). Nuclear magnetic resonance spectroscopy for medical and dental applications: a comprehensive review. Eur. J. Dent. 13, 124–128. 10.1055/s-0039-1688654 31170770 PMC6635960

