# Supplementary material for: Automatic classification of signal regions in 1H Nuclear Magnetic Resonance spectra
Source: Front Artif Intell. 2023 Jan 11;5:1116416. doi: 10.3389/frai.2022.1116416 (PMC9874632; doi:10.3389/frai.2022.1116416)
Supplement: Supplementary file 1 [file Data_Sheet_1.PDF]

Supplementary Material

1 SYNTHETIC SPECTRA GENERATION

Phase and baseline distortions

We added a frequency dependent phase distortion $\Delta\phi = \phi_0 + \phi_1\,\omega$, where $|\phi_0| \leq 6\,rad$ and $|\phi_1| \leq 3.9\,rad$.

To add a baseline distortion, we defined two parameters, b_l and b_r, with a modulus lower than two times the noise level. The baseline distortion is defined as $\Delta b = b_r\,x + b_l\,(1-x)$, where x is a linear function increasing monotonically from 0, at the left border of the spectrum, to 1 at the right border of the spectrum.

Table S1. *Parameters ranges*. In the table are reported the range of values for the parameters of the multiplets included in the synthetic spectra.

Parameter	Ranges
γ	0.5-$7Hz$
$\frac{J}{2\gamma}$	0.5-18
ϕ_0	-6-6
ϕ_1	-3.9-3.9
b_l	-2 noise level-2 noise level
b_r	-2 noise level-2 noise level

2 NETWORK ARCHITECTURE

Table S2. *Network architecture*. In the table are reported the output dimension for each layer, where $None$ is the batch size, S_I is the size of the input, that is the number of spectral points, and the third component is the channel size.

Layer	Output
Inception-like module	$(None, S_I, 64)$
Time-distributed fully connected	$(None, S_I, 64)$
Bi-directional LSTM	$(None, S_I, 512)$
Time-distributed fully connected	$(None, S_I, 512)$
Time-distributed fully connected	$(None, S_I, 512)$
Time-distributed fully connected	$(None, S_I, 64)$
Time-distributed fully connected	$(None, S_I, 8)$
Softmax output	$(None, S_I, 8)$

3 EXPERIMENTAL TEST SET

Table S3. *Experimental test set*. In the table are reported the details of the 10 experimental spectra used to evaluate the model. In the sixth column we listed the number of resonances belonging to the classes of multiplets represented in the training set of the model.

Compound	Solvent	Base frequency (MHz)	Points	PPM range	Multiplets
Quinine	Acetone	400	65536	−3.84-16.19	1 singlet<br>3 doublets<br>1 triplet
Eburnamonina	CDCl3	500	65536	−1.50-14.50	4 singlets<br>2 triplets
Geraniol	CDCl3	400	32768	−4.10-16.45	1 singlet
Camphor	CDCl3	400	32768	−4.10-16.45	7 singlets<br>1 triplet<br>1 quartet
3-Ethyltoluol	DMSO	400	32768	−4.10-16.45	2 triplets<br>1 quartet
Cumol	DMSO	400	32768	−4.10-16.45	2 singlets<br>1 doublet<br>1 septet
L-lactic acid	DMSO	400	32768	−4.10-16.45	3 doublets<br>2 quartets
Naringenin	DMSO	400	32768	−4.10-16.45	3 singlets
Cinchocaine	DMSO	600	32768	−4.19-16.44	2 doublets<br>7 triplets<br>1 quartet<br>1 quintet<br>1 sextet
Quinine	MeOD	400	32768	−4.10-16.45	2 singlets<br>4 doublets<br>5 triplets<br>1 quintet
